# Bronchial erosion and migration of Port-A-Cath, a case report

**DOI:** 10.1016/j.ijscr.2019.06.049

**Published:** 2019-06-26

**Authors:** Mahseeri Mohamad, Tayseer Ahmad Sabbah Al-Tawarah, Mohammad Alqasem Ayed Odeh Aladaileh, Azzam Hunsi Khalaf, Hebah Hisham Hawasheen, Mahmoud Abu-Abeeleh

**Affiliations:** a General Surgery Department, Faculty of Medicine, University of Jordan, P.O Box 13857, Amman 11942, Jordan; b General Surgery Department, Faculty of medicine, Yarmouk University, 21163, Irbid, Jordan

**Keywords:** Central line, Venous access, Migration, Erosion, Port-A-Cath

## Abstract

**Introduction:**

Several possible complications are known to be related to central venous catheters.

**Presentation of case:**

A 20-year-old lady had a catheter (Port-a-Cath) inserted in the left subclavian vein to treat Hodgkin's lymphoma. One year after insertion, on receiving intravenous chemotherapy the patient began coughing. Imaging studies showed the tip of the catheter to be crossing the superior mediastinum into the upper lobe of the right lung. The patient was taken to the operating room and the Port-a-Cath catheter was removed intact.

**Discussion:**

Migration is a rare complication associated with insertion of these lines. Accompanying erosion into an airway is even rarer.

**Conclusion:**

Any unexplained deterioration in the respiratory system should raise suspicion of catheter migration and bronchial erosion.

## Highlights

• Central venous lines are used to obtain a long lasting vascular access for total parenteral nutrition, chemotherapy, as well as obtaining blood samples. They are widely used in hematology/oncology patients.
• Complications of the insertion of Port-A-Catheters may include thrombotic and infectious complications. Catheter migration is relatively a rare complication but potentially life threatening.
• Clinicians should recognize the development of bronchial erosion upon clinical suspicion confirmed by radiological investigations.
• Treatment is tailored at removing the catheter and correcting any possible complication due to migration.

## 1 Introduction

Totally inserted central venous lines are used to obtain long-lasting vascular access for the delivery of total parenteral nutrition, chemotherapy, as well as obtaining blood samples. They are widely used in hematology/oncology patients. They contribute greatly to improving the quality of life for oncologic patients, particularly patients for whom obtaining intravenous access is difficult. Complications of the insertion of Port-A-Catheters may include thrombotic and infectious complications. Catheter migration is relatively a rare complication but potentially life-threatening. The present case study investigates a case of catheter migration and erosion into the bronchial tree one year after insertion. This work has been reported in line with the SCARE criteria [1].

## 2 Report of the case

**Fig. 1:**
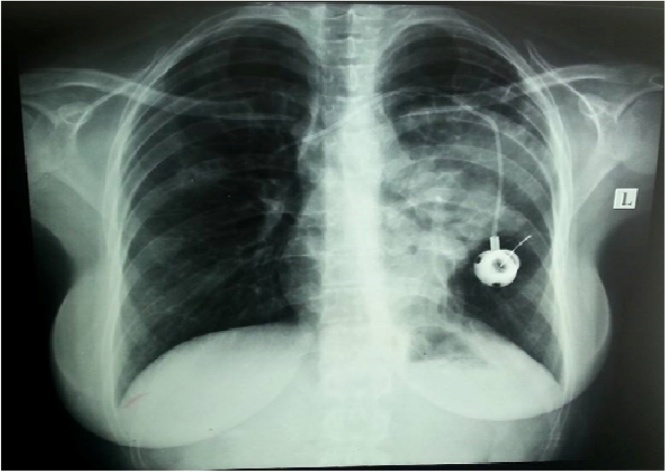
Opacity at the left hemithorax with evidence of effusion.

**Fig. 2:**
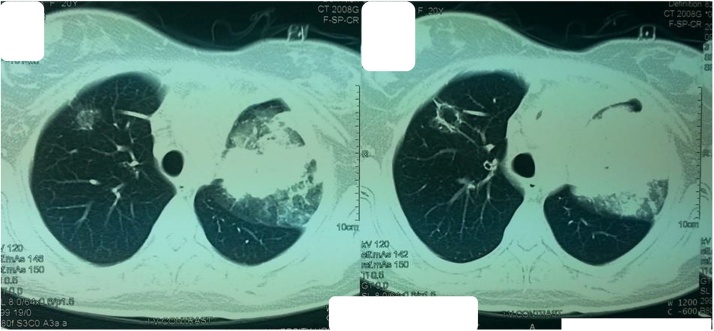
Venous erosion of the port into the right upper lung lobe.

A 20-year-old lady had a catheter (Port-a-Cath) inserted in the right subclavian vein to treat Hodgkin's lymphoma. The catheter was removed a year later due to a nonfunctioning catheter and replaced by a left-sided subclavian vein catheter and received chemotherapy thereafter. She was unavailable for follow-up for three months, she showed after 3 months and continued her chemotherapy, at this time and upon giving the chemotherapy the patient experienced coughing during a routine flush of the catheter that was associated with regurgitation of salty fluid, she denied any chest and neck pain, or shortness of breath. Physical examination disclosed no abnormalities except for a subcutaneous reservoir anchored to the left pectoral muscle. Her ECG Findings were normal as were the results of her hematologic and blood chemistry profiles. A chest radiograph was obtained immediately (Fig. 1), followed by a chest CT scan (Fig. 2) which showed the tip of the catheter seen crossing the superior mediastinum into the upper lobe of the right lung. She was taken to the operating room and under general anesthesia; the Porta- Cath catheter was removed intact. No bleeding occurred. She was discharged the following day.

## 3 Discussion

The totally implanted central venous access (TIAP), one of which is used often is referred to as Port-A-Cath, is a small reservoir attached to a venous catheter and located in subcutaneous tissue. This device began to be used at the beginning of 1980 and currently considered as an essential part of daily clinical practice [2].

Despite that, and as a result of the growing use of intravenous ports, many complications arose. According to reports, the complication percentage varies from 0.4% to 29% [[2], [3], [4], [5]]. Preliminary complications involve malposition, arrhythmia, hematoma, pneumothorax, or embolism, which correlates with the placement technique. While delayed complications comprise thrombosis, infection, skin necrosis, migration, and occlusion [3].

‘Venous port migration’ can be defined as a venous port that is in the wrong position. Implantable catheter tip migration is an uncommon event and there is no clearly defined mechanism for it. By referring to migrations illustrated in the literature, it can be found that most of them are vascular migration with a reported incidence of about 0.9–2% [[3], [4], [5]]. Moeinipour ET. Al illustrated the migration of a central venous catheter to the left internal mammary vein [7], while Ahn et al described a case of port catheter migration to the right axillary vein [6].

The risk factor for vascular migration was examined by other reporters who indicated that there are several factors that lead to catheter migration. These factors include positive pressure ventilation, physical movement, and increased intrathoracic pressure resulted from sneezing, weight lifting, or coughing [2,5]. Caron et al. [4] stated that there are a number of risk factors for perforation associated with the central venous system including the insertion into the left subclavian vein, repeated manipulation of the catheter, and sclerosing fluids administration (e.g. chemotherapy or parenteral nutrition) [8]. Wu et al. mentioned two risk factors for catheter migration including high catheter tip location and lung cancer. Furthermore, Fan et al. indicated that migration rates are considerably higher for male and lung cancer patients. As the most popular site of migration is the internal jugular vein [8].

The symptoms (chest pain or shortness of breath), bronchoscopy, radiographic studies or glucose sputum testing suggest the diagnoses of a patient having this

complication [8]. Through the management of such a challenging complication, simple catheter removal is considered as an unsuitable choice theoretically, because it may lead to serious venous air embolism or possible uncontrollable bleeding into the airway but no reported complication was reported [8].

A Medline search was performed. This report presents the second case of bronchial port migration and erosion into a bronchus.

## 4 Conclusion

Consideration should be given to a catheter migration and erosion during the use of chronic left-sided central venous lines for patients who have a sudden cough or other pulmonary symptoms. Any unjustified deterioration in the respiratory system should raise suspicion of catheter erosion. Clinicians should recognize the development of a bronchial erosion upon clinical suspicion confirmed by radiological investigations. The present case is different from other cases because of the prolonged time from catheter insertion to erosion and that a diagnosis was made before a serious respiratory complication occurred.

## Conflicts of interest

No conflict of interest.

## Sources of funding

No funding.

## Ethical approval

This article is approved by the ethics committee of Jordan University Hospital and IRP of the university of Jordan.

## Consent

Written informed consent was obtained from the patient for publication of this case report and accompanying images. A copy of the written consent is available for review by the Editor-in-Chief of this journal on request.

## Author’s contribution

Mohamad Mahseeri MD.: writing and editing and reviewing.

Tayseer Ahmad Sabbah Al-Tawarah RCSI.: editing.

Mohammad Alqasem Ayed Odeh Aladaileh RCSI.: editing.

Azzam Husni Ragheb Khalaf, RCSI: reviewing.

Hebah hisham hawasheen, RCSI: reviewing.

Professor Mahmoud Abu-abeeleh: reviewing.

## Registration of research studies

N/A.

## Guarantor

Mohamad Mahseei.

## Provenance and peer review

Not commissioned, externally peer-reviewed.
